# Complete Genome Sequence of a Novel Goose Parvovirus Isolated in Sichuan Province, China, in 2016

**DOI:** 10.1128/genomeA.00428-17

**Published:** 2017-06-08

**Authors:** Zhiqi Ge, Shun Chen, Mingshu Wang, Anchun Cheng

**Affiliations:** a Institute of Preventive Veterinary Medicine, Sichuan Agricultural University, Chengdu, Sichuan, China; b Research Center of Avian Disease, College of Veterinary Medicine, Sichuan Agricultural University, Chengdu, Sichuan, China; c Key Laboratory of Animal Disease and Human Health of Sichuan Province, Chengdu, Sichuan, China; d College of Life Science and Technology, Southwest University for Nationalities, Chengdu, Sichuan, China

## Abstract

Here, we report the complete genome sequence of the novel goose parvovirus (NGPV) strain SC16 (NGPV-SC16), which was isolated from Sichuan Province, China, in 2016 and is a cause of the newly emerging beak atrophy and dwarfism syndrome in ducklings and a moderately pathogenic GPV-related parvovirus. The whole genome of strain NGPV-SC16 was 5,109 nucleotides long.

## GENOME ANNOUNCEMENT

Novel goose parvovirus (NGPV), which is a moderately pathogenic GPV-related parvovirus, has been reported in commercial Cherry Valley duck flocks and mule duck flocks in China since 2015 (1–3). The major clinical symptoms of these infected ducks are short bills with protruding tongues and growth retardation, which is why the disease caused by NGPV is called beak atrophy and dwarfism syndrome (BADS). In November 2016, a suspected outbreak of BADS occurred in a Cherry Valley duck farm located in Sichuan Province, China. The clinical signs of the sampled ducks were retarded growth, short beak, and protruding tongue, and anatomical pathology showed that the livers, spleens, kidneys, and lungs of the ducks were tumid. Livers and spleens were sampled as PCR template for whole genome amplification of NGPV Sichuan strain NGPV-SC16. Mainly based on the sequence of the NGPV isolate SDLC01 (GenBank accession no. KT343253.1), five pairs of primers were designed to produce overlapping amplicons spanning the entire viral genome. The amplicons were sequenced by BGI (Chengdu, China), and the full sequence was assembled by SeqMan. The results showed that the genomic sequence of strain NGPV-SC16 was 5,109 nucleotides (nt) in length. The inverted terminal repeats (ITRs) at the 5′ and 3′ ends of the genome were 408 nt and 407 nt, respectively. The NS1 protein was encoded by 1,884 nt and composed of 627 amino acids (aa). The NS2 protein was encoded by 1,356 nt and composed of 451 aa. The VP1 protein was encoded by 2,199 nt and composed of 732 aa. The VP2 and VP3 proteins, which are derived from VP1, were encoded by 1,764 nt and 1,605 nt, respectively, and were predicted to encode 587 and 534 aa, respectively.

Genome sequence alignments demonstrated that strain NGPV-SC16 shared 99.6% identity with strain SDLC01 and 99.3% identity with strain QH15 (GenBank accession no. KT751090.1), which were the highest sequence similarities. The phylogenetic tree analyses based on amino acids of either the NS or VP1 proteins indicated that NGPV-SC16 showed a very close genetic relationship with strains SDLC01 and QH15. Furthermore, phylogenetic analyses also showed that these three strains were distinct from all GPV isolates and Muscovy duck parvovirus and belonged to a distinct GPV-related subgroup. However, NGPV-SC16 still had some genetic variation. Compared with strains SDLC01 and QH15, NGPV-SC16 had two and three extra 14-nt fragments at the 5′ ends of the ITRs, respectively. The ITRs are crucial for viral DNA replication and packaging of parvoviruses (4). So, the significance of these inserts may be worth further investigation.

In conclusion, the whole-genome sequence of NGPV-SC16 will accelerate the acquisition of new knowledge about the evolution and epidemiology of NGPV in China and lay a foundation for the prevention and control of BADS.

### Accession number(s).

The complete genome sequence of NGPV-SC16 has been deposited in GenBank under the accession number KY679174.

## References

[1] Chen H, Dou Y, Tang Y, Zhang Z, Zheng X, Niu X, Yang J, Yu X, Diao Y 2015 Isolation and genomic characterization of a duck-origin GPV-Related parvovirus from Cherry Valley ducklings in China. PLoS One 10:e0140284. doi:10.1371/journal.pone.0140284. 26465143 PMC4605506

[2] Chen S, Wang S, Cheng X, Xiao S, Zhu X, Lin F, Wu N, Wang J, Huang M, Zheng M, Chen S, Yu F 2016 Isolation and characterization of a distinct duck-origin goose parvovirus causing an outbreak of duckling short beak and dwarfism syndrome in China. Arch Virol 161:2407–2416. doi:10.1007/s00705-016-2926-4. 27314945

[3] Yu K, Ma X, Sheng Z, Qi L, Liu C, Wang D, Huang B, Li F, Song M 2016 Identification of goose-origin parvovirus as a cause of newly emerging beak atrophy and dwarfism syndrome in ducklings. J Clin Microbiol 54:1999–2007. doi:10.1128/JCM.03244-15. 27194692 PMC4963502

[4] Qiu J, Cheng F, Yoto Y, Zádori Z, Pintel D 2005 The expression strategy of goose parvovirus exhibits features of both the *Dependovirus* and *Parvovirus* genera. J Virol 79:11035–11044. doi:10.1128/JVI.79.17.11035-11044.2005. 16103154 PMC1193622

